# Lipolysis-Stimulated Lipoprotein Receptor Acts as Sensor to Regulate ApoE Release in Astrocytes

**DOI:** 10.3390/ijms23158630

**Published:** 2022-08-03

**Authors:** Ameziane Herzine, Ghita Sekkat, Sandra Kaminski, Gaetano Calcagno, Sandrine Boschi-Muller, Hela Safi, Catherine Corbier, Sophie Siest, Thomas Claudepierre, Frances T. Yen

**Affiliations:** 1 UR 3998 Animal and Functionality of Animal Products Laboratory, Quality of Diet and Aging Team, University of Lorraine, 54505 Nancy, France; ameziane.herzine@univ-lorraine.fr (A.H.); ghita.sekkat@univ-lorraine.fr (G.S.); catherine.corbier@univ-lorraine.fr (C.C.); thomas.claudepierre@univ-lorraine.fr (T.C.); 2 UR 7300 Stress Immunity Pathogens Laboratory, Faculty of Medicine, University of Lorraine, 54505 Nancy, France; sandra.kaminski@univ-lorraine.fr (S.K.); gaetano.calcagno@univ-lorraine.fr (G.C.); 3 UMR 7365 Molecular Engineering and Articular Physiopathology Laboraotory, Faculty of Medicine, University of Lorraine, 54505 Nancy, France; sandrine.boschi@univ-lorraine.fr (S.B.-M.); hela.safi@umontpellier.fr (H.S.); 4 UR 1122 Gene-Enviornment Interactions in Cardio-Vascular Physiopathology, Faculty of Medicine, University of Lorraine, 54500 Nancy, France; sophie.visvikis-siest@inserm.fr

**Keywords:** lipolysis-stimulated lipoprotein receptor, cholesterol, astroglia, lipoprotein, apolipoprotein E, siRNA knockdown, qPCR, cell culture

## Abstract

Astroglia play an important role, providing de novo synthesized cholesterol to neurons in the form of ApoE-lipidated particles; disruption of this process can increase the risk of Alzheimer’s disease. We recently reported that glia-specific suppression of the lipolysis-stimulated lipoprotein receptor (LSR) gene leads to Alzheimer’s disease-like memory deficits. Since LSR is an Apo-E lipoprotein receptor, our objective of this study was to determine the effect of LSR expression modulation on cholesterol and ApoE output in mouse astrocytes expressing human ApoE3. qPCR analysis showed that siRNA-mediated *lsr* knockdown significantly increased expression of the genes involved in cholesterol synthesis, secretion, and metabolism. Analysis of media and lipoprotein fractions showed increased cholesterol and lipidated ApoE output in HDL-like particles. Further, *lsr* expression could be upregulated when astrocytes were incubated 5 days in media containing high levels (two-fold) of lipoprotein, or after 8 h treatment with 1 µM LXR agonist T0901317 in lipoprotein-deficient media. In both conditions of increased *lsr* expression, the ApoE output was repressed or unchanged despite increased *abca1* mRNA levels and cholesterol production. We conclude that LSR acts as a sensor of lipoprotein content in the medium and repressor of ApoE release, while ABCA1 drives cholesterol efflux, thereby potentially affecting cholesterol load, ApoE lipidation, and limiting cholesterol trafficking towards the neuron.

## 1. Introduction

The role of astrocytes in the physiopathology of the central nervous system (CNS) has been largely overlooked until recently [1]. With their central position linking the blood supply to the neuron, astroglia cells are crucial in maintaining brain homeostasis by providing nutrients to neurons, removing cell debris, and limiting metabolic product accumulation [1]. Astrocytes play a particularly important role in the regulation of brain cholesterol homeostasis [2], which is essential for maintaining the integrity, function, and communication between the different networks of cells that make up the CNS [2]. Astrocytes ensure de novo synthesis of cholesterol, which is secreted in the form of Apolipoprotein (ApoE)-containing nascent HDL-like particles [2,3]. The ATP binding cassette transporter A1 (ABCA1) is required for facilitating transport of intracellular cholesterol to nascent ApoE to form ApoE-containing HDL-like particles, which are secreted for delivery to neurons [2,3].

This lipid-mediated crosstalk may represent a means to ensure bi-directional communication between neurons and glial cells, allowing the latter to adjust the synthesis, efflux, and elimination of cholesterol to meet the needs of neurons [4]. Neuronal cholesterol levels have been correlated with the amyloidogenic pathway and thus production of the amyloidogenic Aβ peptide [5,6], increasing risk of Alzheimer’s disease (AD). Moreover, age-related changes in cholesterol levels have been reported in the hippocampus and cortex [7,8]. Since these two regions are important in learning and memory, it was postulated that this cholesterol homeostasis disequilibrium could lead to increased risk of neurodegeneration and AD. The presence of lipidated ApoE has been shown to be important for astrocyte-mediated Aβ peptide clearance [9,10]. Investigation of the molecular mechanisms involved in the regulation of astrocyte cholesterol metabolism, and thereby cholesterol trafficking, is, therefore, essential for a better understanding of the regulation of this critical and essential process in the brain.

Since ApoE serves as a ligand for lipoprotein receptors, these membrane proteins play key roles in mediating cholesterol dialogue between the neuron and glia. Numerous studies in the literature have focused on the LDL-receptor (LDL-R), the LDL-R related protein 1 (LRP1), and other ApoE receptors from the same family in the regulation of brain cholesterol homeostasis [11], neuronal function, and synaptic plasticity [12,13]. We have identified and characterized another ApoE lipoprotein receptor, LSR (lipolysis-stimulated lipoprotein receptor), which was recently reported to be expressed in the brain at levels higher in glial cells as compared to neurons [14]. LSR, originally identified and characterized in the liver [15], is able to bind ApoB or ApoE-containing lipoproteins [16,17]. Our studies of heterozygote *lsr^+/−^* mice revealed that the dyslipidaemia developed in these mice [17] is associated with significant age-dependent changes in brain cholesterol trafficking [18], including changes in cholesterol distribution [18], and an increase in cortical 24S-OH cholesterol metabolite [19]. Furthermore, cortical cholesterol content was significantly correlated with Aβ-induced difficulties in learning in aged *lsr^+/−^* mice [19], suggesting that changes in cholesterol trafficking due to deficits in LSR can increase the risk of neurodegenerative processes and AD. Targeted deletion of the *lsr* gene in Glast-positive astroglia leads to significant behavioural changes, including memory and olfactory deficits similar to some aspects of AD [20]. Furthermore, changes in the expression of genes related to cholesterol homeostasis in the hippocampus were observed in these mice [20]. Taken together, these in vivo studies clearly show that deficits in *lsr* can lead to significant cognitive deficits correlated with changes in brain cholesterol homeostasis. Since these observations were made in in vivo models, we questioned if the modification of *lsr* expression could directly affect cholesterol metabolism at the cellular level. The objective of this study was, therefore, to investigate the effect of modulating *lsr* expression on astrocyte cholesterol metabolism and ApoE secretion using a cell culture model of immortalized murine astrocytes expressing the human ApoE3 isoform [21].

## 2. Results

### 2.1. Effect of siRNA-Mediated lsr Knockdown on Astrocyte Cholesterol Homeostasis

**Figure 1:**
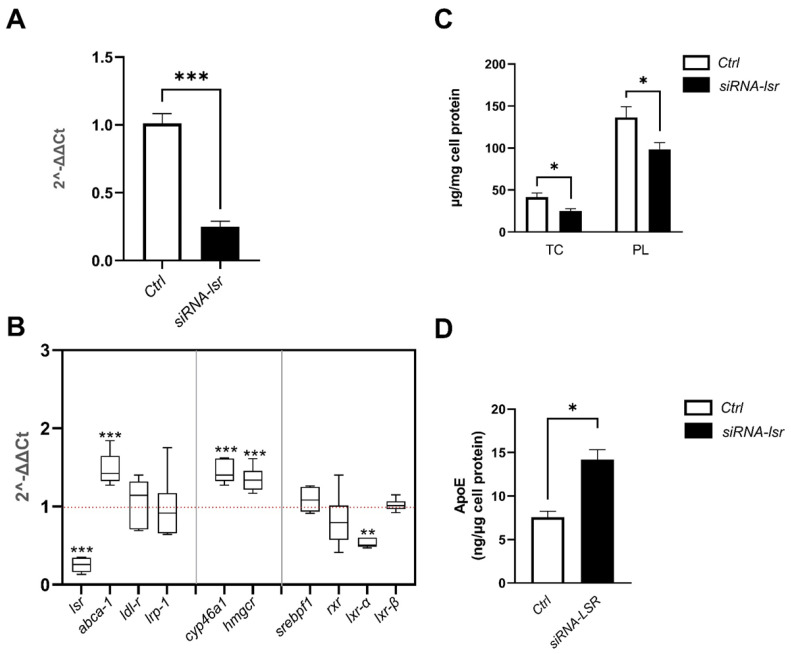
Effect of siRNA-mediated knockdown of *lsr* on cellular cholesterol homeostasis of murine astrocytes expressing human ApoE3. Astrocytes were treated with siRNA targeting *lsr* (siRNA-*lsr*) or siRNA negative control (Ctrl) as described in the Materials and Methods. After 5 days incubation at 37 °C, cells and media were removed and stored for RT-qPCR of target genes, and assays of cell cholesterol and human ApoE in media. (**A**) qPCR analysis of lsr mRNA levels; (**B**) qPCR analysis of target genes indicated; (**C**) cell total cholesterol (TC) and phospholipid (PL) levels; (**D**) ApoE levels secreted in the cell media expressed per mg cell protein. Results are shown as mean ± SEM (*n* = 12 per group). Statistical differences are shown as: * *p* < 0.05, ** *p* < 0.01, *** *p* < 0.001 (abbreviations: *abca1*, ATP binding cassette transporter A1; *cyp46a1*, cytochrome P450 family 46 subfamily A member 1; *hmgcr*, 3-hydroxy-3-methylglutaryl-CoA reductase; *ldl-r*: low-density lipoprotein-receptor; *lrp-1*, LDL-R related protein 1; *lxr*, liver X receptor; *rxr*, retinoid X receptor; *srebpf1*, sterol regulatory element binding protein 1).

siRNA was used to knock down *lsr* expression in a murine astrocyte cell line expressing human ApoE3. siRNA targeting *lsr* (siRNA-*lsr*) or an siRNA-negative control (Ctrl) were transfected into astrocytes. After 5 days of culture, total mRNA was prepared from cell lysates. The RT-qPCR results showed a significant four-fold decrease in *lsr* mRNA levels in cells treated with *lsr* siRNA (*p* < 0.001) as compared to those in Ctrl cells (Figure 1A). This was in line with previous results using the same siRNA, which leads to decreased mRNA and protein levels of LSR in another murine cell line [17].

Having verified *lsr* knockdown, RT-qPCR was next performed on a series of key genes involved in lipid homeostasis (Figure 1B), including cholesterol transport (*abca1*), lipoprotein uptake (*ldl-r* and *lrp-1*), cholesterol synthesis (*hmgcr*), cholesterol metabolism (*cyp46a1*), and transcription factors (*lxr α*, *lxr β*, *srebpf1*, *rxr*). The results revealed that siRNA-*lsr* treatment induced an increase in the expression of *abca1* (1.5-fold, *p* < 0.001), *cyp46a1* (1.4-fold, *p* < 0.001), as well as *hmgcr* (1.4-fold, *p* < 0.001), but also a decrease in the expression of *lxr α* (0.5-fold, *p*< 0.01) when compared to the control group. Non-significant differences between siRNA-*lsr* and Ctrl were observed in *ldl-r*, *lrp-1*, *srebpf1*, *lxr β*, or *rxr* gene expression levels. These results show clearly that *lsr* deficit can lead to changes in mRNA expression of a specific set of genes involved in brain cholesterol homeostasis, including synthesis (*hmgcr*), efflux (*abca1*), or elimination (*cyp46a1*) of cholesterol.

In view of the significant expression changes in genes involved in cholesterol metabolism and turnover, cholesterol was measured in lipid extracts from cells. Cell total cholesterol and phospholipid levels were significantly lower by almost two-fold after siRNA-*lsr* treatment (Figure 1C, *p* < 0.05). In addition, ELISA measurement of human ApoE in the media revealed a two-fold increase in human ApoE3 secreted in the medium of the cells treated with siRNA-*lsr* compared to the Ctrl cells (Figure 1D, *p* < 0.001).

**Figure 2:**
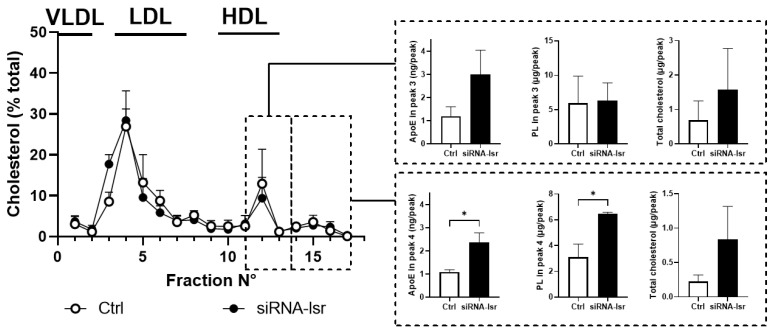
Effect of siRNA-mediated knockdown of *lsr* on lipoprotein profiles in culture media from murine astrocytes expressing human ApoE3. Cell culture media were collected from astrocytes treated with siRNA targeting *lsr* (siRNA-*lsr*) or siRNA negative control (Ctrl) (see Figure 1). The media were pooled, concentrated, and fractionated by gel filtration chromatography as described in Materials and Methods. Fractions were analysed for total cholesterol and ApoE content. **Left** panel: relative cholesterol levels (% of total) in each fraction are shown (mean ± SEM, *n* = 3–4 per group). The fractions corresponding to elution of plasma lipoproteins, VLDL, LDL, and HDL are indicated above the chromatogram. **Right** panel: ApoE, phospholipid, and total cholesterol levels in pooled fractions of the corresponding peaks are indicated (**top right** panel for fractions 11–13; **bottom right** pane for fractions 14–17; *n* = 3–4/group). Statistical differences are shown as: * *p* < 0.05.

To determine if the released ApoE3 was in the lipidated or non-lipidated form, cell culture media were recovered and fractionated by gel filtration chromatography to isolate the different lipoprotein fractions based on size. Total cholesterol levels were determined in each fraction, and the distribution amongst different fractions was calculated as the percentage of the total amount of cholesterol (Figure 2). The results revealed one major peak that eluted in fractions corresponding to the larger plasma lipoproteins VLDL and LDL, which most likely represents lipoproteins in the foetal bovine serum that is also in the media. Two smaller peaks were identified corresponding to plasma HDL. Since astrocytes are known to produce HDL-like particles, ApoE, phospholipids, and total cholesterol levels were determined in these two fractions, shown in the right panels for each corresponding peak. Both ApoE and PL levels were significantly increased in the smaller HDL-like peak in media from cells following siRNA-induced knockdown of *lsr*, indicating that the ApoE3 in the media (Figure 1D) was in the lipidated form.

Taken together, these results indicate that a siRNA-mediated decrease in *lsr* expression levels led to increased cholesterol and ApoE levels in cell media. In addition, analysis of lipoprotein profiles suggests increased ApoE lipidation, which would be consistent with the observed increase in cholesterol transporter *abca1* gene expression.

### 2.2. Effect of Culture Media Lipoprotein Content on lsr Expression in Astrocytes

**Figure 3:**
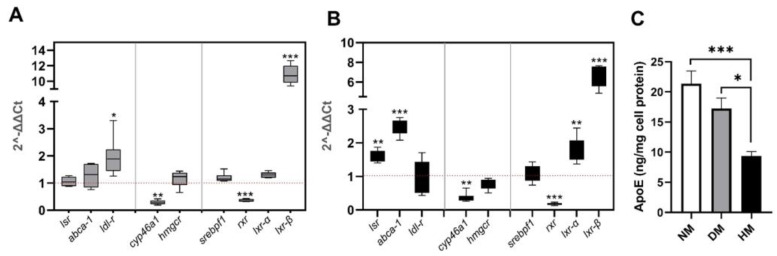
Effect of lipoprotein-depleted or high lipoprotein containing media on expression of *lsr*, genes related to cholesterol homeostasis, and ApoE levels in astrocytes. Murine astrocytes expressing human ApoE3 were incubated in the presence of normal growth media (NM), lipoprotein-depleted media (DM), or high lipoprotein containing media (HM). After 5 days incubation at 37 °C, cells and media were removed and stored for RT-qPCR of target genes, and determination of human ApoE in media by ELISA. Results of qPCR analysis of target gene levels are shown in cells incubated with DM (**A**) or HM (**B**). (**C**) Human ApoE levels in the cell media expressed as ng ApoE secreted in the media per mg cell protein. Results are shown as mean ± SEM (*n* = 6 per group). Statistical differences are shown as * *p* < 0.05, ** *p* < 0.01, *** *p* < 0.001 (for (**A**,**B**), as compared to control cells in NM; for (**C**), as indicated).

As we previously showed that free fatty acids can modify LSR activity [16], we questioned if lipoprotein levels in the media could also regulate *lsr* expression in astrocytes. Lipoprotein-deficient serum was prepared by ultracentrifugation at density (*d*) 1.25 g/mL. The lower lipoprotein-deficient *d* > 1.25 g/mL fraction was used at concentrations equivalent to that of (10%, *v*/*v*) foetal bovine serum to prepare astrocyte-lipoprotein-depleted medium (DM). The cells were incubated in DM and, therefore, in the absence of lipoproteins, or in NM, for 5 days, the same period of time as that for the experiment using siRNA *lsr* in Figure 1. Comparative qPCR analysis of DM using NM as a control revealed no significant changes in astrocyte *lsr* expression (Figure 3A). On the other hand, an increase in *ldl-r* mRNA levels was observed (two-fold, *p* < 0.05) in cells incubated in DM as compared to those in NM, consistent with previous studies by other investigators showing DM-induced upregulation of this receptor [22]. Increased expression of the transcription factor *lxr β* (11-fold, *p* < 0.001) was also observed. Decreases in *cyp46a1* (0.3-fold, *p* < 0.01) and *rxr* (0.4-fold, *p* < 0.001) mRNA levels were observed, and no detectable differences were observed in *abca1*, *hmgcr*, *srebpf1*, and *lxr α* mRNA levels in cells incubated in DM.

To determine the effect of high levels of lipoprotein in the media, the total lipoprotein fraction (*d* < 1.25 g/mL) obtained following ultracentrifugation of foetal bovine serum was used to supplement normal media (NM). This high lipoprotein containing media (HM) contained lipoprotein levels that were two-fold higher than that of NM. Coomassie blue staining after SDS-PAGE of the total lipoprotein fraction revealed a majority of ApoA1; ApoE was present but only at very low levels (data not shown).

Incubation of astrocytes in this lipoprotein-enriched HM medium led to increased expression of both *lsr* (1.6-fold, *p* < 0.001) and *abca1* (2.5-fold, *p* < 0.001), as well as the transcriptional factors *lxr α* (1.8-fold, *p* < 0.001) and *lxr β* (6.6-fold, *p* < 0.001) (Figure 3B), as compared to the levels in cells in NM. On the other hand, significantly lower mRNA levels of *cyp46a1* (0.4-fold, *p* < 0.01) and *rxr* (0.18-fold, *p* < 0.001) were observed. There were no significant differences for *ldl-r*, *hmgcr*, or *srebpf1* expression levels in cells incubated in HM as compared to NM, suggesting an absence of changes in cholesterol synthesis. This indicated, therefore, that high levels of lipoprotein in the HM media led to increased expression not only of the *abca1* cholesterol transporter but also of *lsr*.

Measurement of human ApoE3 released by astrocytes into the media revealed that ApoE levels decreased more than two-fold (*p* < 0.001, compared to NM; *p* < 0.05 compared to DM) in the media in astrocytes incubated with HM media (Figure 3C, solid bar). On the other hand, there were no statistically significant changes in human ApoE levels in cells following incubation with DM as compared to those incubated in NM (Figure 3C, grey bar), which was consistent with the lack of expression changes in *lsr*, *hmgcr*, and *abca1*.

In Figure 1, we observed that increased *abca1* expression led to increased cholesterol efflux and ApoE production following siRNA-mediated *lsr* knockdown. While both *abca1* mRNA levels and human ApoE3 concentration in the media did not change in cells incubated in DM as compared to those in NM, a significant increase in *abca1* gene expression was observed in astrocytes incubated with HM. However, under these same HM conditions, the ApoE levels in the media did not parallel *abca1* expression and were in fact significantly lower by at least two-fold. This would suggest the presence of an additional controlling factor of ApoE production and/or secretion. The increased *lsr* expression in cells incubated in HM suggested that LSR levels are regulated by the presence of lipoproteins in the media. LSR may, therefore, act as a lipid sensor and could participate in the retrocontrol of ApoE levels that could be independent of ABCA1. Since the LXR/RXR pathway has been shown by other investigators to be involved in the regulation of cholesterol efflux [23,24], we questioned if the regulation of *lsr* was also driven by this transcription factor.

### 2.3. Effect of LXR Agonist T0901317 on lsr Expression in Astrocytes

**Figure 4:**
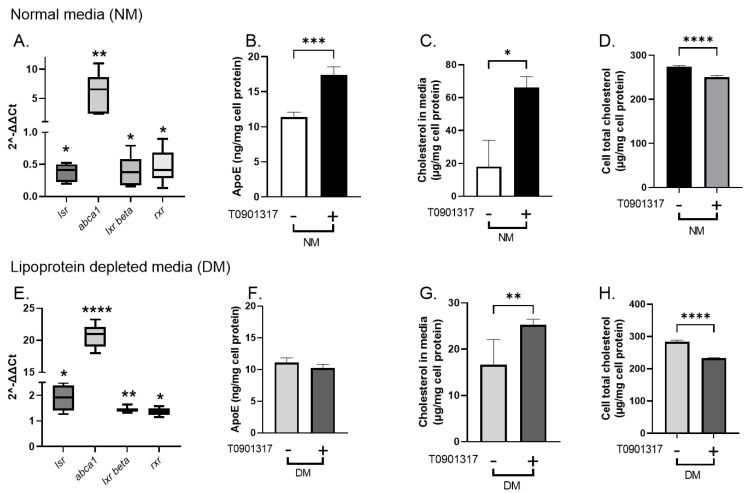
Effect of T0901317 on *lsr* expression in murine astrocytes expressing human ApoE3. Astrocytes were incubated 8 h at 37 °C in the absence (−) or presence (+) of 1 µM T0901317 in complete media (NM, (**A**–**D**)) or in lipoprotein-depleted media (DM, (**E**–**H**)). After 8 h incubation at 37 °C, cells and media were removed and stored for analysis. Results of fold-expression changes in cells treated with T0901317 as compared to control cells (absence of T0901317) following qPCR analysis of target gene levels are shown in (**A**,**E**) for NM and DM media, respectively. ApoE and total cholesterol levels in the cell media expressed per mg cell protein and cell lipid extracts are indicated in (**B**–**D**) for NM and (**F**–**H**) for DM. Results are shown as mean ± SEM (*n* = 6). Statistical differences are shown as: * *p* < 0.05, ** *p* < 0.01, *** *p* < 0.001, **** *p* < 0.0001 as compared to control cells.

To test this, we used T0901317, a specific LXR agonist that has been shown by other investigators to increase *abca1* expression [23,24]. Astrocytes were treated 48 h after plating with 1 µM T0901317 in normal media NM (Figure 4A–D). Preliminary dose and time response studies showed that 8 h incubation at 37 °C was sufficient to detect T0901317-induced *abca1* transcription (data not shown). The cells were washed and recovered to analyze the gene expression by RT-qPCR. A comparative analysis was performed using NM (absence of T0901317) as the control. Treatment of cells with T0901317 in NM led to statistically significant decreased levels of *lsr* mRNA (0.37-fold, *p* < 0.05), as well as those of *lxr β* (0.4-fold, *p* < 0.05) and *rxr* (0.5-fold, *p* < 0.05). The mRNA *abca1* levels were increased more than six-fold (*p* < 0.001), confirming the LXR agonist effect on this gene involved in cholesterol transport reported by other investigators [23,24] (Figure 4A). Analysis of ApoE media content revealed higher levels in astrocytes treated with LXR agonist in NM as compared to controls in the absence of T0901317 (Figure 4B, 1.5-fold, *p* < 0.001). To calculate cholesterol output, the basal amount of total cholesterol in NM before incubation was subtracted from the measured total cholesterol in NM after incubation. The calculated difference showed increased media cholesterol content after T0901317 treatment in NM (Figure 4C, 3.8-fold, *p* < 0.05). A significant parallel decrease in cell total cholesterol levels was also observed (Figure 4D). Fractionation of concentrated cell media by gel filtration chromatography revealed increased cholesterol levels in the larger HDL-like peak (peak 3, Supplementary Figure S1A,B) and increased ApoE in both the small and smaller HDL-like peak (peaks 3 and 4, Supplementary Figure S1A,B) after T901317. Taken together, the T0901317-induced increase in cholesterol efflux, higher lipidated ApoE levels, and decreased *lsr* levels were consistent with the results obtained following siRNA-*lsr* knockdown (Figure 1 and Figure 2).

Astrocytes were treated with the same concentration of T0901317 and for the same time period but this time in the absence of lipoproteins. Here, a comparative analysis of mRNA levels was performed using DM alone (absence of T0901317) as controls. The absence of lipoproteins in the media (DM) led to a T0901317-induced increase in *lsr* (1.9-fold, *p* < 0.05), *lxr β* (1.4-fold, *p* < 0.01) and *rxr* (1.4-fold, *p* < 0.05) mRNA levels. Again, the *abca1* levels were increased following T0901317 treatment (20-fold, *p* < 0.0001) (Figure 4E). However, despite the increased *abca1*, and in contrast to the previous experiment using NM (Figure 4A,B), there was no difference in media ApoE levels in astrocytes incubated in DM in the absence or presence of T0901317 (Figure 4E,F).

Cholesterol levels in the media were increased following T0901317 treatment of cells in DM (Figure 4G, 1.5-fold, *p* < 0.01), consistent with increased *abca1* expression and decreased cell cholesterol content (Figure 4H). The cholesterol:ApoE molar ratio was calculated for the cells incubated in lipoprotein-deficient DM since these values represent only those produced by the astrocytes. The ratio was found to increase significantly from 138.1 ± 23.6 (control) to 226.2 ± 22.9 (T0901317, *p* < 0.05) in astrocytes, suggesting increased lipidation of ApoE particles in the presence of the LXR agonist in DM. Lipoprotein fractionation showed changes in the cholesterol distribution after T0901317 treatment. An apparent increase in cholesterol in the first HDL-like peak was observed without any evident changes in ApoE distribution between the different lipoprotein peaks (Supplementary Figure S1C,D), which is consistent with the increased cholesterol:ApoE molar ratio.

These results demonstrate that T0901317 induced an increase in *abca1* and cholesterol efflux and ApoE lipidation in DM media and increased lsr expression, but without changes in ApoE levels in the media.

### 2.4. LSR and ABCA1 Localization in Astrocytes

**Figure 5:**
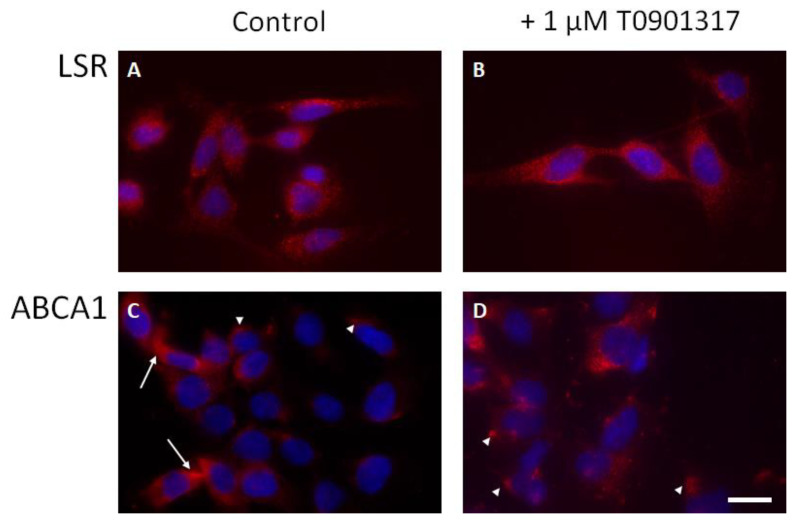
LSR and ABCA1 localization in astrocytes. LSR (**A**,**B**) and ABCA1 (**C**,**D**) protein levels were detected by immunofluorescence (red) in astrocytes grown in normal growth media (NM) in absence (**A**,**C**) or presence (**B**,**D**) of LXR agonist treatment (8 h, 1 µM T0901317). Co-labelling with DAPI to stain cell nuclei (blue) is shown in all panels (Scale bar = 10 µm). Arrows show strongly labelled cells; arrowheads show cells with discrete patchy or focal staining.

Immunocytochemistry, performed without permeabilization, revealed a punctuated staining of LSR protein, homogeneously distributed on the cell membrane of the astrocytes (Figure 5A). LXR agonist treatment did not appear to affect LSR distribution at the membrane, as seen in Figure 5B. For ABCA1 transporter, the staining revealed few cells strongly labeled (arrows, Figure 5C), while other cells exhibited a discrete patchy or focal staining (arrowheads, Figure 5C). LXR agonist appeared to affect ABCA1 localization, where labeling of ABCA1 became more robust and was observed in all cells (Figure 5D).

## 3. Discussion

**Figure 6:**
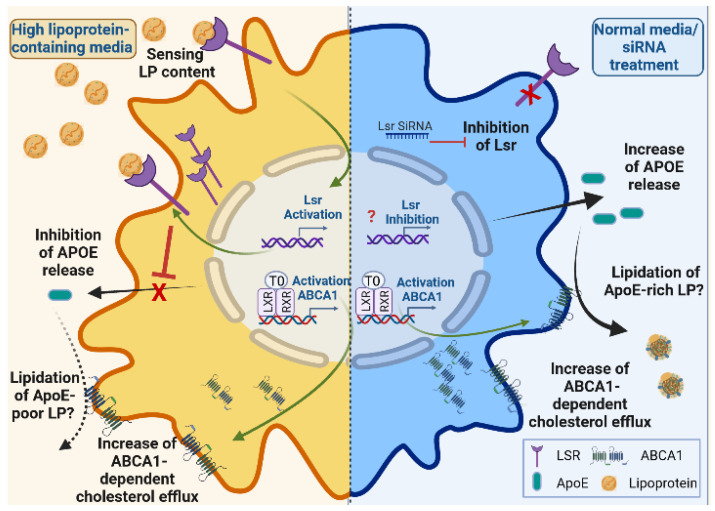
Working hypothesis of LSR effect on ApoE output in astrocytes. Our data show that high extracellular levels of lipoprotein (HM) stimulate *lsr* expression and decrease ApoE levels, suggesting a negative retro control of ApoE release from astrocytes, thus modulating ABCA1-mediated lipidation of lipoproteins. On the other hand, *lsr* reduction (siRNA) leads to release of lipidated ApoE and cholesterol into the medium. Transcriptional regulation of *lsr* by the LXR/RXR pathway depends on the lipoprotein content in the medium, while LXR upregulation of *abca1* is not. We propose that the decoupling of cholesterol and ApoE release could influence the composition and lipidation of LP released by astrocytes. (LP = lipoprotein). Created with Biorender.com (accessed on 19 July 2022).

This is the first study demonstrating a direct link between astrocyte *lsr* expression and ApoE release in a murine astrocyte model. Our results show that siRNA-mediated *lsr* knockdown leads to changes in cholesterol metabolism, ultimately increasing cholesterol efflux, ApoE production, and its lipidation in cultured astrocytes (Figure 6). *lsr* expression was upregulated when cells were exposed to high levels of lipoprotein in the cultured media, resulting in lower levels of ApoE in the media and thus changes in lipoprotein composition. ABCA1 is important for cholesterol efflux and is regulated through the LXR pathway [23,24]. Here, we observed that this LXR activation of *abca1* expression occurred under both NM and DM conditions, while LXR-agonist T0901317 mediated changes in *lsr* expression that were dependent on the presence of lipoproteins in the culture media. This suggests the presence of other regulatory mechanisms independent of the LXR pathway, mediating cell response to extracellular lipid levels by increasing *lsr* expression, thereby repressing ApoE production.

siRNA-mediated knockdown of *lsr* led to an increase in both cholesterol de novo synthesis and transport, as indicated by increased *hmgcr* and *abca1* expression levels, as well as increased human ApoE3 secreted by the astrocytes into the media. In the astrocyte, HMG-CoA reductase is the rate-limiting enzyme in cholesterol de novo synthesis [2]. Cholesterol is then secreted with ApoE in the form of nascent or discoidal HDL-like lipoproteins with the aid of the ABC transporters. ABCA1 plays a key role in cholesterol trafficking, including cholesterol efflux and lipidation of ApoE, as demonstrated by Hirsch-Reinshagen et al., who reported decreased cholesterol levels in the cerebrospinal fluid in *abca1*-KO mice, as well as reduced cholesterol and decreased ApoE secretion in extracellular media in *abca1*-deficient astrocytes [25]. Here, siRNA *lsr* knockdown led to increased ApoE secretion into the media. Since these murine astrocytes produce human ApoE, we were able to directly measure the amount secreted from these cells using a human ApoE3-specific ELISA. Lipoprotein fractionation revealed two major and one minor peaks, which represents both lipoproteins from foetal bovine serum (FBS) in NM, as well as lipoproteins produced by astrocytes. Previous literature indicates that astrocytes produce nascent and discoidal HDL-like particles [26,27], but we cannot completely rule out that a proportion of the larger lipoprotein fraction may have been produced by astrocytes. Indeed, the lipoprotein profile in cells incubated with DM revealed significant levels of cholesterol in larger LDL-like lipoproteins (Supplementary Figure S1C). Nevertheless, siRNA-mediated *lsr* knockdown did lead to significant amounts of ApoE3 in the smaller HDL peak, which indicates that the majority of ApoE was in the lipidated form.

Despite increased *hmgcr* expression, the cell cholesterol levels were actually lower following siRNA-mediated *lsr* knockdown (Figure 1). Together, this would suggest that *lsr* knockdown induced increased cholesterol efflux towards the extracellular media, providing additional insight in the decreased intracellular lipids observed following Nile red staining of brain sections from aged heterozygote *lsr^+/−^* [18]. Although no difference in total cholesterol levels was detected in these mice, these analyses were based on total homogenates rather than specific cell types. Interestingly, the expression of *cyp46a1* was increased as well, which may have contributed to the observed lower cell cholesterol levels. Cyp46A1 is the enzyme responsible for the conversion of cholesterol to 24S-OH cholesterol, the cholesterol metabolite that crosses the blood–brain barrier for excretion by the liver. Cholesterol synthesis is decreased in *cyp46a1*-deficient mice, suggesting that both pathways are linked, which would be consistent with the observations here suggesting increased cholesterol synthesis and metabolism [28]. The levels of *cyp46a1* are normally low in astrocytes and microglia but can be increased in the case of injury or neurodegenerative pathology [29,30]. Increased *cyp46a1* following *lsr* knockdown in astrocytes is also consistent with the higher levels of cortical 24S-HC reported in aged heterozygote *lsr^+/−^* mice that exhibit increased memory deficits following intra-cerebroventricular injection of the oligomeric soluble form of the Aβ peptide [19].

Neither *ldl-r* nor *lrp-1* expression levels in astrocytes were modified after siRNA-mediated *lsr* knockdown, showing that the subsequent changes in expression of genes involved in cholesterol were directly dependent on *lsr* and not on these receptors from the LDL-R family. Moreover, no changes in expression of *srebpf1* were observed, which is a key transcription factor involved in the regulation of *ldl-r* expression.

Further, *lsr* expression was significantly up-regulated when high levels of lipoprotein were introduced into the media (Figure 3B, HM). We previously showed that free fatty acids can modify LSR activity [16]. This is the first study to demonstrate that, in astrocytes, *lsr* expression could be influenced by lipoprotein content. Contrary to the condition in which *lsr* was low, cholesterol synthesis and metabolism genes *hmgcr* and *cyp46a1* were either unchanged or repressed, respectively. This would suggest that the increased influx of lipids, potentially via the LSR pathway because of increased *lsr* expression, served as a signal to decrease de novo cholesterol synthesis and metabolism. Despite this, there was a significant increase in *abca1*, suggesting higher cholesterol efflux, which may have been due to increased levels of *lxr β*, a transcription factor known to activate *abca1* transcription [31]. Previous studies using other cell types have shown that *abca1*-mediated cholesterol efflux and ApoE secretion are regulated by lipoprotein content in the media [2,25]. Although ApoE expression has been shown to be activated by *lxr β* [24], ApoE levels in the media were found in this study to be significantly lower, which we postulate as being due to a repressor effect following activation of *lsr*. Further investigation is required to determine the specific lipid, lipoprotein fraction, or sub-fraction that leads to upregulation of *lsr* expression observed here, which may provide insight into the regulatory mechanisms involved.

On the other hand, no changes in *lsr* expression were observed when astrocytes were incubated in absence of lipoproteins (DM). Up-regulation of the *ldl-r* was observed, consistent with the well-established effect of DM on this receptor by Goldstein and Brown [22]. Under these conditions, no changes in *abca1* were observed, nor was there a significant decrease in ApoE levels in the medium, suggesting no increased efflux of cholesterol or ApoE production from the astrocytes.

The use of T0901317 as an LXR agonist proved to be a strong activator of *abca1* expression in both normal (5-fold) and in lipoprotein-depleted medium (15-fold). The LXR/RXR transcriptional pathway regulates cholesterol efflux by targeting *abca1* and ApoE genes [2,31]. These nonsteroidal receptors form heterodimers with RXR, and, in the presence of oxysterols, the LXR/RXR heterodimer recruits coactivators, which induce the expression of target genes, including *abca1* and ApoE. The LXR family includes two isoforms: *lxr α*, which is prominently expressed in the liver and other tissues essential for peripheral lipid metabolism, such as the kidneys, small intestine, spleen, and adipose tissue. *lxr β* is found predominantly in the liver and brain. 

The effects of the LXR agonist in NM (Figure 4A–D) were very similar to those observed following siRNA-mediated knockdown of *lsr* expression (Figure 1). In both conditions in which *lsr* expression was reduced, *abca1*-mediated cholesterol transport, ApoE production, and lipidation were increased. The lipoprotein profile was slightly different in the two conditions, where a higher portion of cholesterol was found in the smaller dense HDL (Supplementary Figure S1A), but this may be due to the shorter incubation time (8 h vs. 5 days). With a lesser number of cycles of lipoprotein synthesis, secretion, and reuptake, the lipoprotein profile at 8h may have been more representative of newly synthesized particles secreted into the media, while that obtained after 5 days represents lipoproteins following significant remodelling by ABCA1, ApoE, and other factors. Nevertheless, these data confirm through two different treatments to decrease *lsr* expression that the absence of LSR significantly increases cholesterol output and ApoE production and lipidation.

The results showed that LXR agonist had different and opposite effects on *lsr* depending on the culture medium, with *lsr* down-regulation in NM and *lsr* up-regulation in lipoprotein-depleted medium DM. This indicates that additional regulatory mechanisms yet to be identified exist and that the *lsr* gene was not under unidirectional control by the T0901317 LXR agonist. In lipoprotein-depleted medium (DM), *lsr* was increased in the presence of T0901317. Under these conditions, ApoE was not increased and remained steady even if *abca1* was strongly up-regulated and cholesterol efflux was increased. This was similar to the repression of ApoE release following HM-induced increase in lsr expression (Figure 3B,C), which would lead us to suggest that LSR has the ability to repress ApoE release and thus significantly influence ABCA1-driven cholesterol efflux and ApoE lipidation and modify the composition or type of lipoproteins released into the media (Supplementary Figure S1).

At the protein level, we observed a clear effect of LXR on ABCA1 localization at the astrocyte membrane (Figure 5D). The increase in large patches of ABCA1 may correspond to the focal adhesion area [32] or cholesterol-rich non-raft structures [33]. ABCA1 protein expression changes in the presence of T0901317 appeared to correlate with the qPCR analyses showing increased *abca1* gene expression in the presence of this LXR agonist. In the LXR agonist experiments using NM, LSR expression on cell membranes appeared similar, with a robust punctuated staining in all astrocytes (Figure 5 for NM). Since the cells were not permeabilized in order to selectively stain LSR and ABCA1 on the plasma membrane surface, newly synthesized proteins may not have been detected under these experimental conditions. While not the focus of this paper, it would be interesting to determine if LSR cellular localization could be affected by lipid composition in the medium.

Based on these results, we propose that, in astrocytes, LSR acts as a sensor of lipid content in the medium and repressor of ApoE release, while ABCA1 drives cholesterol efflux. The combination of both resulted in a modulation of lipoprotein composition by limiting the ApoE content in secreted lipoproteins. This sensor ability of LSR would be expected to dramatically affect cholesterol supply to neurons as those cells take up cholesterol from ApoE-rich lipoproteins [34]. LSR repression on ApoE release would lead to ApoE-poor lipoprotein output, which would limit cholesterol trafficking towards neurons and affect their cholesterol load [35]. LSR might, therefore, be a key dispatcher of glia-derived cholesterol, directing it toward ApoE rich/poor lipoproteins depending on extracellular lipid levels. In line with this hypothesis, it has been suggested that ApoA1-containing lipoproteins could serve as a lever to reduce the cholesterol load in the CNS [36,37]. This would be consistent with the upregulation of *lsr* expression by the ApoA1-rich HM fraction. One of the limitations of this study lies in the fact that the results were obtained using a homogenous cell culture model of murine astrocytes expressing human ApoE3. To determine how the changes in ApoE and cholesterol efflux induced by LSR would affect neuronal uptake of these modified lipoproteins and, ultimately, glia-neuron cholesterol communication requires additional investigation using a combination of astrocytic and neuronal cultures.

To conclude, these data bring to light new evidence demonstrating an important role of astroglial LSR in cell cholesterol metabolism. These results reinforce our working hypothesis that cognitive deficits in *lsr^+/−^* or glial *lsr* KO mice may be a direct result of lsr-deficit-induced changes in brain cholesterol homeostasis [18,19,20]. *lsr* expression is dependent upon lipoprotein levels in the extracellular media and is directly correlated with lipidated ApoE output and, therefore, lipoprotein composition. We, therefore, propose astroglial-LSR as a potential target of interest for a better understanding of regulating cholesterol homeostasis in the aging brain, which is essential for preservation of cognitive function.

## 4. Materials and Methods

### 4.1. Cell Culture

Cell culture studies were performed at the Bioavailability–Bioactivity (Bio-DA) platform. Immortalized murine astrocytes expressing human apoE3 were kindly provided by D.M. Holtzman [21]. Cells were maintained in advanced DMEM containing 10% heat-inactivated FBS, 100 mM glutamine, 50 mg/mL of geneticin [21] (Gibco, Thermofisher, Illkirch-Graffenstaden, France). For experiments, cells were seeded in 6-well plates (50,000 cells/well for siRNA, lipoprotein enrichment or depletion, and 200,000 cells/well for LXR agonist), and then treated at 48 h as described below. The number of different wells (*n*) from at least 3 different culture preparations are specified for each treatment. All assays were performed in duplicate or triplicate.

#### 4.1.1. siRNA Treatment

After washing with PBS, cells were treated with *lsr*-targeted or with negative control siRNA (Ambion, Thermofisher) using Interferin (Polyplus, Illkirch-Graffenstaden, France) transfection agent as previously described [17], followed by incubation at 37 °C for five days (*n* = 12 per group).

#### 4.1.2. Lipoprotein Enrichment (HM) or Depletion (DM) Treatment

Foetal bovine serum (Gibco) was adjusted to *d* 1.25 g/mL using sodium bromide (Merck Sigma, St. Quentin Fallavier, France), overlaid with *d* 1.21 g/mL sodium bromide, then centrifuged at 45,000× *g* for 48 h. The top *d* < 1.25 g/mL phase containing all lipoproteins was removed. The lipoprotein fraction (LP) and the d > 1.25 g/mL lipoprotein-deficient serum fractions were then dialyzed extensively in PBS to remove the sodium bromide. Fraction volumes were measured before and after in order to determine those that were equivalent to that of the original foetal bovine serum. Apolipoprotein composition was verified by Coomassie Blue staining after separation on 10% SDS-PAGE (20 µg protein/lane).

For treatments, astrocytes were treated 48 h after plating with lipoprotein-depleted media (DM) containing lipoprotein-depleted serum (equivalent to 10% *v*/*v* foetal bovine serum) or with high lipoprotein containing media (HM) containing the equivalent of 2-fold of lipoproteins. Untreated cells incubated in normal growth media (NM) served as controls. Cells were incubated at 37 °C for 5 days (*n* = 6 per group).

#### 4.1.3. LXR Agonist Treatment

Forty-eight hours after plating, cells were washed in PBS (Gibco, Waltham, MA, USA), then incubated for 8 h at 37 °C in either normal media (NM) containing 10% (*v*/*v*) foetal bovine serum, or lipoprotein-depleted media (DM) (amount corresponding to 10% foetal bovine serum) in the absence or presence of 1 µM T0901317 (Merck-Sigma, St. Louis, MO, USA). Control cells were treated with the same volume of vehicle alone (DMSO, Merck-Sigma), corresponding to <1% volume (*n* = 6 per group).

#### 4.1.4. Preparation of Samples for Analyses

After incubations, cell culture medium was removed, centrifuged to remove any floating cells or debris (3000× *g*, 10 min, 4 °C), and stored at −20 °C for later analyses. Cells were washed with PBS. For real-time PCR analysis, cell pellets were prepared, washed with PBS, and snap-frozen for total RNA extraction. Cell lysates were prepared using ice-cold RIPA (Thermofisher, Waltham, MA, USA) buffer, followed by centrifugation at 13,000× *g* for 10 min at 4 °C to remove cell debris. Protein concentration of the supernatants was determined using a bicinchoninic acid (BCA) protein assay kit (Pierce, Thermofisher).

### 4.2. qPCR (RNA Extraction, RT and Real-Time PCR)

Total RNA was extracted using TRI reagent (Merck Sigma), according to the manufacturer’s instructions. RNA quantity and purity were estimated by a Nanodrop ND-2000 spectrophotometer (Thermo Scientific; Villebon-sur-Yvette, France), and the samples with a 260/280 nm ratio ≥1.8 were used for subsequent analyses. Reverse transcription was performed using 1 μg of RNA in a final volume of 20 µL, including 0.5 µL of random primers (Invitrogen, Cergy Pontoise, France), 1 μL of 10 mM dNTP mix (Invitrogen), in RNase-free water. After denaturation of RNA samples at 65 °C for 5 min, 4 μL of buffer (5×), 2 μL of 0.1 mM DTT, 1 μL of Superscript II reverse transcriptase (Invitrogen), and 1 μL of RNase OUT (Invitrogen) were added. Samples were homogenized and transcribed according to the following conditions: 25 °C for 10 min, 42 °C for 50 min, and 70 °C for 15 min (Eppendorf Master Cycler, Eppendorf, Montesson, France). The cDNA obtained was used as a template for the PCR array using the PowerUP SYBER Green master mix (Applied Biosystems, Thermofisher) with the following final concentrations in a 25 μL final volume: 1 × Master Mix, 100 nM forward and reverse primers, 0.4 ng/μL cDNA. Real-time PCR was performed in the Fast Real-Time PCR system (Applied Biosystems) using the following thermal cycling conditions: 5 min denaturation at 95 °C, 42 cycles of 15 s at 95 °C, 1 min at 60 °C, and a final dissociation step. The primer specificity was determined based on the presence of a single peak in the melting curve. Primers (Eurogentec, Seraing, Belgium) used for the target genes are shown in Table 1. Phosphoglycerate kinase 1 (*pgk1*) was used as reference gene. Quantitation was performed by the 2^−∆∆Ct^ method.

### 4.3. ApoE and Lipid Assays

Human ApoE3 levels secreted in cell culture media were measured using an ELISA kit specific for human ApoE (Merck-Sigma).

Lipids were extracted from frozen cell pellets using the Folch method (Chloroform:methanol, 2:1, *v*/*v*). The chloroform phase containing lipids was removed and evaporated, and the dried lipids were redissolved in isopropanol containing 10% Triton X-100. Cholesterol levels were directly measured in cell culture media (NM) or after concentrating media (DM) 10-fold using Millipore concentration filters (10,000 cut-off). Cholesterol and phospholipids were determined using enzymatic kits (Amplex red cholesterol assay, ThermoFisher; LabAssay Phospholipids assay, Wako, Neuss, Germany) according to the manufacturer’s instructions.

### 4.4. Immunocytochemistry

Astrocytes cultures were fixed with 4% paraformaldehyde (Merck-Sigma) for 20 min. Nonspecific sites were blocked by incubating 1 h at room temperature in 30% Cas-Block (Invitrogen) in PBS (Gibco). Cells were then incubated overnight at room temperature, with primary antibodies: rabbit LSR (1/200, HPA007270, Sigma) or mouse ABCA1 (1/200, MAB10005, Merck-Millipore, Fontenay-sous-Bois, France) prepared in 3% Cas-Block solution. Cells were not permeabilized in order to allow a specific staining of the LSR receptor and ABCA1 transporter at the membrane. Cells were incubated 1 h at room temperature with the appropriate secondary antibodies. Alexa 555-anti mouse (1/500, A21422) and Alexa555-anti rabbit (1/500, A21430) conjugated secondary antibodies were acquired from Molecular Probes (Invitrogen). For all staining, nuclei were visualized by incubation with DAPI (1/1000, Merck-Sigma) for 10 min at room temperature. Finally, the slides were mounted on glass slides using Fluoromount-G Electron Microscopy Sciences, Hatfield, PA, USA) and left at room temperature overnight, protected from light. Slides were then examined using a fluorescence microscope (Axio Imager.D2, Zeiss, Oberkochen, Germany) equipped with an Orca flash4.0 LT+ digital camera (Hamamatsu Photonics, Hamamatsu, Japan). Image captions were performed using identical settings (intensity, contrast, exposure) for every culture condition. This allowed us to perform qualitative study of LSR and ABCA1 localization at astrocyte membrane depending on the treatment.

### 4.5. Lipoprotein Profiles

Media from cell culture were pooled (6–12 mL) and then concentrated to approximately 500 µL using Amicon 15 mL centrifugal filter units with cutoff of 10 K kDA, following manufacturer’s instructions (Merck-Millipore, Fontenay-sous-Bois, France). Concentrated samples (62.5 µL) were applied at 1 mL/min to a Bio SEC-5 column (Agilent, Courtaboeuf, France) equilibrated in phosphate buffered saline in a UHPLC system (Ultimate^TM^ 3000 BioRS System, Thermo Fisher Scientific, UMS2008/US40 IBSLor technical platform). Fractions (500 µL) were collected and analysed for total cholesterol and phospholipids using Amplex red cholesterol assay or Lab Phospholipid assay (WAKO), respectively, or for ApoE levels using ELISA of pooled fractions. Runs were performed with human serum samples on the same column under identical conditions to obtain the elution profile of human serum lipoproteins. Three major lipoprotein classes were identified and are indicated in Figure 2: VLDL (fractions 1–2), LDL (4–6), and HDL (fractions 9–11).

### 4.6. Statistical Analysis

The Kolmogorov–Smirnov test was used to verify normal distribution. Since all variables followed a normal distribution, parametric statistics were used to evaluate significance. For results obtained after real time PCR analyses, fold changes of mRNA samples were compared to corresponding controls and tested for statistical significance (*p* < 0.05) using the Relative Expression Software Tool 2009 (REST Version 2.0.13, Qiagen, Hilden, Germany). Student’s *t*-test was used to compare treated cells with controls for results obtained for ApoE and lipid levels or two-way ANOVA for comparing ApoE levels in cells treated with high lipoprotein containing media (HM) or lipoprotein-depleted media (DM) (Figure 3).

Boxplots (Figure 1B, Figure 3A,B and Figure 4A,E) were obtained using REST software tool, where (+) represents the mean value, the middle line represents the median, the lower (Q1) and upper (Q3) lines in the bar represent the 25% and 75% quartile, respectively. While the upper and lower lines represent the observations outside the 9–91 percentile range, data falling outside of Q1 and Q3 range are plotted as outliers of the data.

## Figures and Tables

**Table 1:** Sequence of primers used for qPCR.

Gene	Forward Primer (5′-3′)	Reverse Primer (5′-3′)
*abca1*	CAACCCCTGCTTCCGTTATCCAA	GAGAACAGGCGAGACACGATGGAC
*cyp46a1*	GGCTAAGAAGT TGGTCCTGTTGTAAGA	GGTGGACATCAGGAACTTCTTGACT
*hmgcr*	CCCCACATTCACTCTTGACGCTCT	GCTGGCGGACGCCTGACAT
*ldl-r*	TGGCTATACCTACCCCTCAAGACAG	GATCCCGGAAAGAGACGGAT
*lrp-1*	CGAGAGCCTTTGTGCTGGATGA	CGGATGTCCTCAATGAGGG
*lsr (total)*	AGTAATACACTCCACTGTCTCCCCAG	CAGGAGAATCACCATCACAGGAA
*nr1h3 (lxr α)*	AGGAGTGTCGACTTCGCAAA	CTCTTCTTGCCGCAGTTT
*nr1h2 (lxr β)*	GCTCTGCCTACATCGTGGTC	CTCATGGCCCAGCATCTT
*pgk1*	GAGCCTCACTGTCCAAACTA	CTTTAGCGCCTCCCAAGATA
*rxr*	CAAACATGGGGCTGAACC	GCCCAC-CCACAAGAGTGA
*srebpf1*	GGTCCAGCAGGTCCCAGTTGT	CTGCAGTCTTCACGGTGGCTC

## Data Availability

Data are contained within the article.

## Supplementary Materials

The following supporting information can be downloaded at: https://www.mdpi.com/article/10.3390/ijms23158630/s1.
