# Supplementary material for: Lipolysis-Stimulated Lipoprotein Receptor Acts as Sensor to Regulate ApoE Release in Astrocytes
Source: Int J Mol Sci. 2022 Aug 3;23(15):8630. doi: 10.3390/ijms23158630 (PMC9368974; doi:10.3390/ijms23158630)
Supplement: Supplementary file 1 [file ijms-23-08630-s001.zip › ijms-1820230-supplementary.pdf]

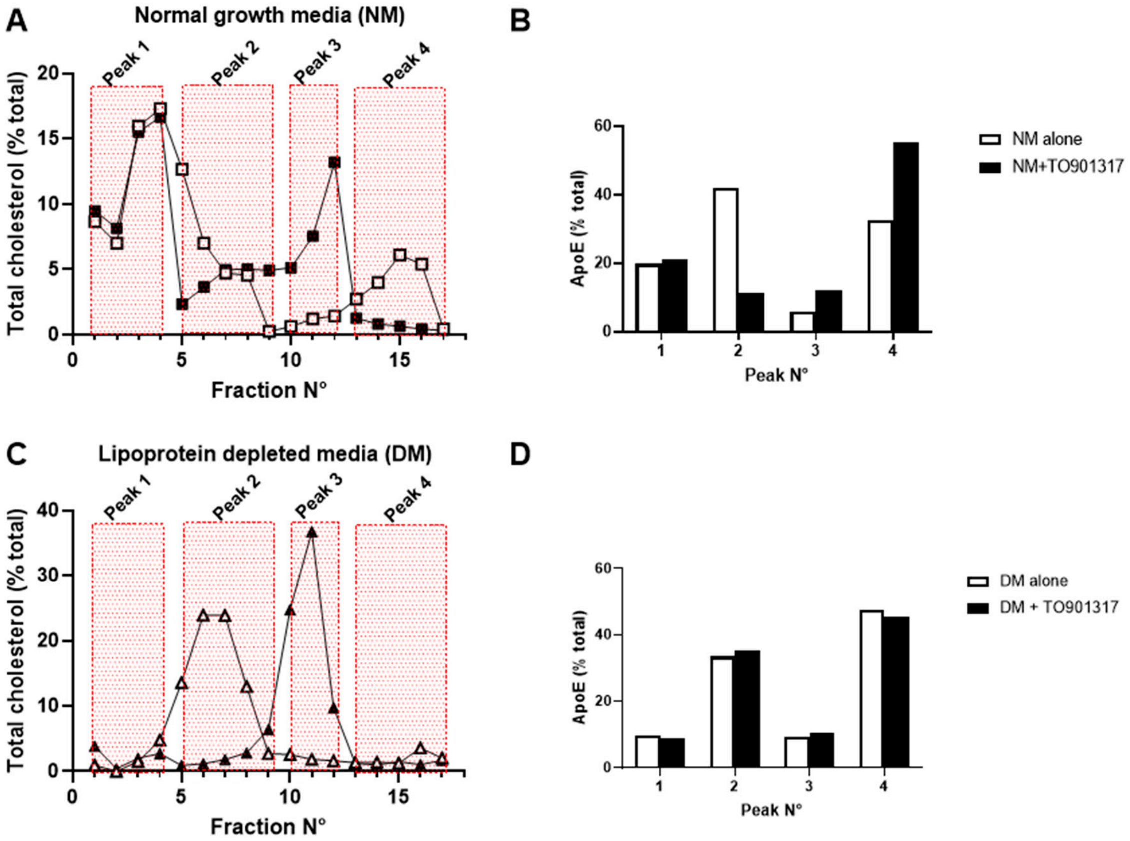

Supplementary Figure S1. Lipoprotein profiles in cell culture media after treatment of astrocytes with T0901317. Cells were incubated 8h at 37°C in the absence (open symbols) or presence (closed symbols) of 1 μM T0901317 (see **Figure 4)**. Cell culture media was collected, concentrated and fractionated by gel filtration chromatography as described in Materials and Methods. A and C) Relative cholesterol levels (% of total) in each fraction are shown (means are shown for $n = 2$ per group). B and D) Relative ApoE levels (% of total) are shown for pooled fractions as indicated (means are shown for $n = 2$ per group).
